# Zika Virus Seropositivity in 1–4-Year-Old Children, Indonesia, 2014

**DOI:** 10.3201/eid2409.180582

**Published:** 2018-09

**Authors:** R. Tedjo Sasmono, Rama Dhenni, Benediktus Yohan, Paul Pronyk, Sri Rezeki Hadinegoro, Elizabeth Jane Soepardi, Chairin Nisa Ma’roef, Hindra I. Satari, Heather Menzies, William A. Hawley, Ann M. Powers, Ronald Rosenberg, Khin Saw Aye Myint, Amin Soebandrio

**Affiliations:** Eijkman Institute for Molecular Biology, Jakarta, Indonesia (R.T. Sasmono, R. Dhenni, B. Yohan, C.N. Ma’roef, K.S.A. Myint, A. Soebandrio);; UNICEF Indonesia, Jakarta (P. Pronyk);; University of Witwatersrand School of Public Health, Johannesburg, South Africa (P. Pronyk);; Universitas Indonesia Medical School, Jakarta (S.R. Hadinegoro, H.I. Satari);; Cipto Mangunkusumo Hospital, Jakarta (S.R. Hadinegoro, H.I. Satari);; Ministry of Health of the Republic of Indonesia, Jakarta (E.J. Soepardi);; Centers for Disease Control and Prevention, Atlanta, Georgia, USA (H. Menzies, W.A. Hawley);; Centers for Disease Control and Prevention, Fort Collins, Colorado, USA (A.M. Powers, R. Rosenberg)

**Keywords:** serology, PRNT, Indonesia, Zika virus, viruses, vector-borne infections, prevalence, seroprevalence, plaque-reduction neutralization, flavivirus

## Abstract

We assessed Zika virus seroprevalence among healthy 1–4-year-old children using a serum sample collection assembled in 2014 representing 30 urban sites across Indonesia. Of 662 samples, 9.1% were Zika virus seropositive, suggesting widespread recent Zika virus transmission and immunity. Larger studies are needed to better determine endemicity in Indonesia.

Zika virus, first isolated in 1947 (*1*), is a flavivirus phylogenetically related to dengue virus (DENV) that is, like DENV, also transmitted by *Aedes* mosquitoes. Because of the epidemic that swept through the Americas in 2016, Zika virus infection is known to cause microcephaly, as well as other congenital defects and Guillain-Barré syndrome (*2*).

Zika virus has long been known to be endemic in Southeast Asia (*3*,*4*), but laboratory confirmation of infection can be challenging. Acute infections are often asymptomatic. In those who are symptomatic, viral RNA typically persists in blood <7 days and in urine <10 days after symptom onset, limiting the usefulness of nucleic acid testing (*5*). Zika virus antibody cross-reacting with DENV can confuse results of tests conducted in regions where the viruses co-circulate (*6*). Virus-specific neutralization assays can more accurately detect and measure Zika virus antibody, but because of their complex requirements, these tests have seldom been used in epidemiologic studies (*7*).

Acute Zika virus cases have been reported in Indonesia (*8*), Singapore (*9*), Malaysia (*10*), Vietnam (*11*), and Thailand (*12*). However, little is known about Zika virus prevalence in the region. Limited retrospective testing of archived specimens collected from clinically ill patients in Thailand (*12*) and Cambodia (*13*) suggest that incidence in these countries is low. However, given the limited number of samples tested and lack of confirmatory testing in these studies, information on prevalence and distribution is challenging to assess. Likewise, little is known about the prevalence and geographic distribution of Zika virus in Indonesia, the biggest country in Southeast Asia.

DENV and chikungunya virus, also transmitted by *Aedes* mosquitoes, are endemic throughout Indonesia, suggesting the ecologic conditions exist for Zika virus transmission as well. An estimated 80% of the population in Indonesia is infected with >1 DENV by the age of 10 years (*14*). In our study, we assessed Zika virus seroprevalence among healthy 1–4-year-old children to determine the prevalence and distribution of Zika virus in Indonesia.

## The Study

We used serum samples collected during October–November 2014 for a previous population-based, cross-sectional cluster survey conducted to assess DENV seroprevalence; in the study, 3,312 samples were collected from 1–18-year-old children in 30 urban districts in 14 provinces of Indonesia (*14*). In our study, we assessed only the children 1–4 years (range 12–59 months) of age because these children were least likely to have cross-reactive DENV antibodies. Ethics clearance was obtained from the Health Research Ethics Committee of the Faculty of Medicine, Universitas Indonesia, and the US Centers for Disease Control and Prevention (CDC; Atlanta, Georgia, USA).

Plaque reduction neutralization tests (PRNTs) that could differentiate Zika virus neutralizing antibodies from those produced in response to DENV infection were adapted from protocols developed by the CDC (Technical Appendix). The challenge virus used in the PRNT was Zika virus JMB-185, acquired from a patient in 2014 (*8*). Convalescent serum from this same patient was used as a PRNT positive control. We subjected all specimens to 2 tiers of testing by PRNT_90_ (i.e., a PRNT in which serum samples suppressing >90% of challenge virus were considered positive for neutralizing antibody). In the first tier, we tested serum samples diluted 1:10. Samples that suppressed >90% of Zika virus PFUs were considered potentially positive for Zika virus antibodies because DENV-specific antibodies, if present, could have cross-reacted and neutralized Zika virus. We then subjected the specimens considered potentially positive to a second PRNT_90_, in which we tested serum samples against Zika virus and all 4 DENV serotypes (Technical Appendix). Specimens that tested positive for Zika virus neutralizing antibody and negative for DENV neutralizing antibody by PRNT_90_ were classified as Zika virus seropositive, as were specimens that had Zika virus PRNT_90_ titers >4-fold higher than all DENV PRNT_90_ titers. We categorized specimens as flavivirus seropositive when Zika virus neutralizing antibodies were present but at titers <4-fold higher than any DENV neutralizing antibody titer (Technical Appendix Table). We also tested a subset of samples for Japanese encephalitis virus antibody by PRNT_90_; none of the samples tested had a titer >20, and none of the sample classifications were changed after testing.

In the initial PRNT_90_ screening, we detected possible Zika virus antibody in 73 (11.0%) of the 662 serum samples (Table). Of these, 72 had a sufficient volume to undergo second-tier testing; 60 (83.3%) of 72 samples were Zika virus seropositive, and 12 (16.7%) were flavivirus seropositive. Serum samples from 11 of 14 provinces were Zika virus seropositive, and the collections from the provinces ranged from ≈4.5% seropositive (North Sumatra, Banten, East Kalimantan) to >18% seropositive (Central Java, Jambi; Figure). Overall, Zika virus seroprevalence in the 1–4-year-old cohort was 9.1% (95% CI 3.95%–11.01%).

**Table:** Seropositivity of 1−4-year-old urban children for Zika virus and other flaviviruses, by province, Indonesia, October–November 2014*

Province	Serologic status, % (no. positive samples/total samples)		
	Suspected Zika virus seropositive†	Confirmed Zika virus seropositive‡	Flavivirus seropositive§
Aceh	0 (0/22)	0 (0/22)	0 (0/22)
North Sumatra	9.1 (2/22)	4.5 (1/22)	4.5 (1/22)
West Sumatra	18.2 (4/22)	13.6 (3/22)	4.5 (1/22)
Jambi	18.2 (4/22)	18.2 (4/22)	0 (0/22)
Lampung	8.7 (2/23)	8.7 (2/23)	0 (0/23)
Banten	4.4 (2/45)	4.4 (2/45)	0 (0/45)
DKI Jakarta	10.6 (7/66)	10.6 (7/66)	0 (0/66)
West Java	11.1 (17/153)	8.5 (13/153)	2.0 (3/153)
Central Java	20.5 (18/88)	18.2 (16/88)	2.3 (2/88)
East Java	11.7 (13/111)	9.0 (10/111)	2.7 (3/111)
Bali	0 (0/22)	0 (0/22)	0 (0/22)
East Kalimantan	4.5 (1/22)	4.5 (1/22)	0 (0/22)
South Sulawesi	0 (0/22)	0 (0/22)	0 (0/22)
Southeast Sulawesi	13.6 (3/22)	4.5 (1/22)	9.1 (2/22)
All provinces	11.0 (73/662), 95% CI 5.34–13.32	9.1 (60/662), 95% CI 3.95–11.01	1.8 (12/662), 95% CI 0.23–3.35

*DENV, dengue virus; PRNT_90_, plaque reduction neutralization test with neutralization defined as >90% reduction in challenge virus PFUs. †Serum samples that neutralized >90% of the challenge virus at a 1:10 dilution on initial Zika virus PRNT_90_ screening. ‡Serum samples that neutralized Zika virus only or had a PRNT_90_ titer >4-fold higher for Zika virus than for any DENV. §Serum samples that neutralized Zika virus and DENV and had a PRNT_90_ titer for Zika virus that was <4-fold higher than that for any DENV.

**Figure:**
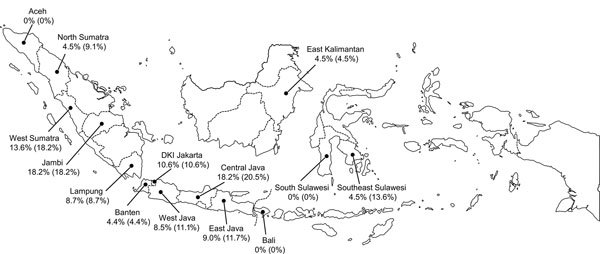
Geographic distribution of Zika virus–seropositive 1−4-year-old children, Indonesia, October–November, 2014. The values listed for each province indicate the percentage of serum samples confirmed Zika virus seropositive (percentage serum samples suspected to be Zika virus seropositive). Samples suspected to be Zika virus positive were those that were positive on initial Zika virus PRNT_90_ (plaque reduction neutralization test with neutralization defined as >90% reduction in challenge virus PFUs) screening when using a 1:10 serum sample dilution. Serum samples confirmed as Zika virus seropositive were those that neutralized Zika virus only or had a PRNT_90_ titer for Zika virus that was >4-fold higher than the PRNT_90_ titer for any DENV.

Our assessment, involving use of the PRNT_90_, which is highly specific for Zika virus antibodies, indicates widespread, recent Zika virus infection in much of western and central Indonesia. Our criterion for confirmed Zika virus antibodies (i.e., PRNT_90_ titer for Zika virus >4-fold higher than that for any DENV in the same specimen) is the international standard. In just 2% (12/662) of specimens, we could not determine whether the antibodies were Zika virus or DENV specific. When using the more conservative criterion of only classifying a sample as positive for Zika virus antibodies if no DENV-specific neutralizing antibodies are detected, the number of Zika virus antibody–positive samples decreases by only 6, leaving 54 samples still classified as Zika virus seropositive. Further evidence for the validity of the PRNT_90_ was that DENV neutralizing antibody–positive samples were negative for the presence of Zika virus neutralizing antibodies across a range of titers (R.T. Sasmono, unpub. data).

Although our data provide some evidence regarding geographic distribution, no information is presented regarding a specific threshold titer associated with clinical illness or a correlation between geography and titer. Further studies involving larger sample sets would be necessary to address these topics. The single age group, relatively small number of specimens, and limited number of sites, particularly from eastern parts of the country, do not give a comprehensive picture of endemicity throughout Indonesia. The small numbers of specimens available from most localities did not enable accurate estimation of the proportional differences between localities. We could perform PRNT_90_ with samples from the remaining cohort (the 5–18-year-olds), but we expect higher percentages of nonspecific flavivirus seropositivity in the samples from this older age group.

## Conclusions

Much has been published on epidemic Zika virus, but little is known about the effect of Zika virus in endemic areas. Determining the prevalence of Zika virus in Indonesia can provide clues to its potential long-term public health significance in endemic settings. Mild or asymptomatic infection is common, and confusion with dengue during diagnosis probably accounts for how long Zika virus was unrecognized in Indonesia and other areas of Southeast Asia. Besides the need to better evaluate Zika virus incidence and distribution, a high priority for future investigations will be determining the extent of Zika virus–related birth defects. If, like other flaviviruses, a primary Zika virus infection results in lifelong immunity, infections during childhood could reduce a person’s risk for infection later in life and thus the incidence of Zika virus–related birth defects. This knowledge provides clues for understanding future patterns of Zika virus transmission in the Americas.

Technical Appendix Description of methods and plaque reduction neutralization test 90% titers for Zika virus and dengue viruses 1−4 of samples from 1−4-year-old children (n = 72), Indonesia, October–November, 2014.
