# Supplementary material for: Zika Virus Seropositivity in 1–4-Year-Old Children, Indonesia, 2014
Source: Emerg Infect Dis. 2018 Sep;24(9):1740–3. doi: 10.3201/eid2409.180582 (PMC6106422; doi:10.3201/eid2409.180582)
Supplement: Technical Appendix — Description of methods and plaque reduction neutralization test 90% titers for Zika virus and dengue viruses 1−4 of samples from 1−4-year-old children (n = 72), Indonesia, October–November, 2014. [file 18-0582-Techapp-s1.pdf]

Article DOI: https://doi.org/10.3201/eid2409.180582

Zika Virus Seropositivity in 1–4-Year-Old Children, Indonesia, 2014

Technical Appendix

Materials and Methods

Cell Lines

The BHK-21 cells (American Type Culture Collection, Manassas, VA, USA) used in plaque reduction neutralization tests (PRNTs) were grown and maintained in RPMI (Roswell Park Memorial Institute) medium supplemented with 10% fetal bovine serum (FBS), 2 mM L-glutamine (Thermo Fisher Scientific, Waltham, MA, USA), and 1× antimicrobial-antimycotic drug solution (100 U/mL penicillin, 100 μg/mL streptomycin, 0.25 μg/mL amphotericin B; Thermo Fisher Scientific). The Vero cells (CCL-81, American Type Culture Collection) used for production of challenge viruses were grown in minimum essential medium supplemented with 5% FBS, 1× antimicrobial-antimycotic drug solution, and 2 mM L-glutamine. Cells were incubated in a controlled and humidified 37°C incubator with 5% CO_2 supplementation.

Serum Samples

Serum samples were derived from a dengue seroprevalence study involving 1–18-year-old urban children in Indonesia (*1*). Serum samples were heat-inactivated for 30 minutes at 56°C before use. A convalescent serum sample from the patient infected with Zika virus strain JMB-185 was used as the Zika virus–positive serum control. For the dengue virus (DENV) PRNTs, DENV–positive antibody controls were obtained from healthy volunteers who tested positive by DENV PRNTs. The Zika virus–negative antibody control was obtained from a healthy volunteer who had previously tested negative by Zika virus PRNT, and the DENV–negative antibody control was obtained from another healthy volunteer who had previously tested negative by DENV PRNT, as detailed below.

Challenge Viruses

The challenge virus used in the Zika virus PRNT was Zika virus strain JMB-185, isolated in Indonesia in 2014 as described previously (*2*). The complete genome of Zika virus JMB-185 has been reported, and on the basis of phylogenetic analysis, this isolate was classified as belonging to the Asian lineage (*3*). The DENV challenge viruses were the parental strains of the Sanofi Pasteur (Lyon, France) recombinant CYD vaccine viruses (*4*), namely DENV-1 strain PUO-359, DENV-2 strain PUO-218, DENV-3 strain PaH881/88, and DENV-4 strain 1228. The DENV strains were kindly shared by Sanofi Pasteur. The source of the DENV strains and their use in DENV neutralization assays have been described and accepted by the World Health Organization (*5*).

Challenge virus stocks were produced in Vero cells (seeded at 5×10^5 cells/flask 3 days before infection) by infecting cells at a multiplicity of infection (MOI) of 0.001 viruses/cell for Zika virus, DENV-1, DENV-2, and DENV-4, and an MOI of 0.01 viruses/cell was used for DENV-3. After the virus adsorption period of 90 minutes at 37°C with 5% CO_2, the inoculums were aspirated and replaced with fresh minimum essential medium containing 5% FBS, 1× antimicrobial-antimycotic drug solution, and 2 mM L-glutamine. Flasks were incubated in a 37°C incubator with 5% CO_2 supplementation until detectable signs of cytopathic effect (CPE) were seen on days 4–8. On the day of harvest, cell culture medium containing virus was collected and centrifuged for 10 minutes at 1,500 × *g* and 4°C to remove cell debris. FBS and sorbitol (Sigma, St. Louis, MO, USA) were added to the clarified supernatant to a final concentration of 20% FBS and 10% (w/v) sorbitol to stabilize the virus. Virus stocks were aliquoted, flash-frozen, and transferred to a –80°C freezer for long-term storage.

Plaque Reduction Neutralization Test (PRNT)

The PRNT protocol was adapted from the US Centers for Disease Control and Prevention (Atlanta, Georgia, USA) and Sanofi Pasteur protocols (*4*,*6*,*7*). BHK-21 cells were seeded at 2.5×10^5 cells/well in 12-well tissue culture plates (Corning, Corning, NY, USA) and incubated for 2 days in a 37°C incubator with 5% CO_2. In each batch of samples tested, anti-Zika virus–positive and anti-Zika virus–negative serum samples were included as controls. Medium only was added to control wells. RPMI medium supplemented with 2% FBS, 2 mM L-glutamine, and 1×

antimicrobial-antimycotic drug solution was used as serum diluent for serum samples, controls, and challenge virus.

For initial Zika virus $PRNT_{90}$ (PRNT with neutralization defined as $\geq$90% reduction in challenge virus PFUs) screening, unknown serum samples were diluted 1:5 in the wells of 96-well plates. A suspension of 60 PFUs of Zika virus strain JMB-185 in 60 µL was then mixed 1:1 with diluted serum samples and incubated for 1 h at 37°C to enable neutralization to occur. In addition, virus-only controls and two 10-fold serially diluted (1:10 and 1:100) virus-only controls were included to determine the $PRNT_{90}$ cutoff. After the neutralization step, culture medium was then aspirated from wells of 12-well plates containing BHK-21 cell monolayers. Without delay, the serum-virus suspensions were then inoculated onto designated wells of 12-well cell culture plates. Plates were incubated for 1 h at 37°C with agitation every 20 min to enable nonneutralized Zika virus to infect BHK-21 cell monolayers. At the end of virus adsorption period, the inoculum was then aspirated and each well was overlaid with 1 mL of 1% carboxymethylcellulose (CMC) RPMI medium supplemented with 2% FBS, 1× antimicrobial-antimycotic drug solution, 0.4% $NaHCO_3$, 2.5 mM HEPES, and 0.5% dimethyl sulfoxide. Plates were incubated at 37°C with 5% CO_2 for 5 days. After the incubation period, the CMC overlay medium was removed from wells and the cell monolayers were fixed with 3.7% formaldehyde solution for 30 minutes. Plates were washed with tap water and stained with 1% crystal violet staining solution for 5 minutes. Plates were finally rinsed with tap water and air-dried. The presence of Zika virus–infected cells was indicated by the formation of viral plaques, marked by a clear area of detached cells. Serum samples that neutralized $\geq$90% of the challenge virus in the initial Zika virus screening were suspected Zika virus seropositive.

For the Zika virus–DENV $PRNT_{90}$ combination format with endpoint titrations, a similar assay setup was prepared, with the addition of DENV challenge viruses (DENV-1–4) and six 2-fold dilutions of the serum samples. Serum samples were initially diluted 1:5 in the first wells of 96-well plates followed by six 2-fold serial dilutions. Suspensions of Zika virus, DENV-1, DENV-2, DENV-3, and DENV-4 adjusted to 60 PFUs/60 µL were prepared. Diluted serum samples were mixed with an equal volume of each challenge virus, and after a 1-h neutralization step, the serum-virus suspensions were inoculated onto BHK-21 cell monolayers in 12-well plates. Inoculums were aspirated, and CMC medium was overlaid, followed by a 5-day

incubation period at 37°C with 5% CO_2. The cell monolayers were fixed with 3.7% formaldehyde solution and stained with 1% crystal violet staining solution.

The neutralization titer ($PRNT_{90}$ titer) of the serum samples was defined as the reciprocal of the highest test serum dilution for which the virus infectivity was reduced by 90% when compared with the average plaque count of the challenge virus controls. Plaques for all 6 serial dilutions of the serum samples were counted to ensure a dose-response reduction. The dilution factor of 2, generated by the addition of an equal volume of challenge virus to the diluted serum sample, was included in the final calculation of the neutralization titers (i.e., 1:10, 1:20, 1:40, 1:80, 1:160, and 1:320). Hence, the theoretical lower limit of quantitation of the assay is a $PRNT_{90}$ titer of 10 (reciprocal of the dilution). Serum samples that neutralized Zika virus only or had a Zika virus $PRNT_{90}$ titer $\geq$4-fold greater than the $PRNT_{90}$ titer of any DENV serotype were considered confirmed Zika virus seropositive. Serum samples that neutralized Zika virus and any DENV and had a Zika virus $PRNT_{90}$ titer <4-fold greater than the $PRNT_{90}$ titer of any DENV were considered flavivirus seropositive.

References

1. Prayitno A, Taurel A-F, Nealon J, Satari HI, Karyanti MR, Sekartini R, et al. Dengue seroprevalence and force of primary infection in a representative population of urban dwelling Indonesian children. PLoS Negl Trop Dis. 2017;11:e0005621. PubMed http://dx.doi.org/10.1371/journal.pntd.0005621

2. Perkasa A, Yudhaputri F, Haryanto S, Hayati RF, Ma'roef CN, Antonjaya U, et al. Isolation of Zika virus from febrile patient, Indonesia. Emerg Infect Dis. 2016;22:924–5. PubMed http://dx.doi.org/10.3201/eid2205.151915

3. Yudhaputri FA, Trimarsanto H, Perkasa A, Yohan B, Haryanto S, Wiyatno A, et al. Genomic characterization of Zika virus isolated from Indonesia. Virology. 2017;510:248–51. PubMed http://dx.doi.org/10.1016/j.virol.2017.07.026

4. Timiryasova TM, Bonaparte MI, Luo P, Zedar R, Hu BT, Hildreth SW. Optimization and validation of a plaque reduction neutralization test for the detection of neutralizing antibodies to four serotypes of dengue virus used in support of dengue vaccine development. Am J Trop Med Hyg. 2013;88:962–70. PubMed http://dx.doi.org/10.4269/ajtmh.12-0461

5. Guirakhoo F, Arroyo J, Pugachev KV, Miller C, Zhang ZX, Weltzin R, et al. Construction, safety, and immunogenicity in nonhuman primates of a chimeric yellow fever-dengue virus tetravalent vaccine. J Virol. 2001;75:7290–304. PubMed http://dx.doi.org/10.1128/JVI.75.16.7290-7304.2001

6. Lindsey HS, Calisher CH, Mathews JH. Serum dilution neutralization test for California group virus identification and serology. J Clin Microbiol. 1976;4:503–10. PubMed

7. Lindsey NP, Staples JE, Powell K, Rabe IB, Fischer M, Powers AM, et al. Ability to serologically confirm recent Zika virus infection in areas with varying past incidence of dengue virus infection in the United States and U.S. Territories in 2016. J Clin Microbiol. 2017;56:1–6. PubMed http://dx.doi.org/10.1128/JCM.01115-17

Technical Appendix Table. Endpoint titration results of combination Zika virus–DENV $PRNT_{90}$ of serum samples from 1–4-year-old children, Indonesia, October–November, 2014

		$PRNT_{90}$ titer					
No.	Sample ID	Zika virus	DENV-1	DENV-2	DENV-3	DENV-4	Serostatus
1	02–1-08	80	<10	<10	<10	<10	Zika virus seropositive
2	02–1-10	40	<10	40	<10	<10	Flavivirus-seropositive
3	03–1-03	80	<10	<10	<10	<10	Zika virus seropositive
4	03–1-08	160	<10	<10	<10	<10	Zika virus seropositive
5	03–1-12	80	<10	<10	<10	<10	Zika virus seropositive
6	03–1-13	40	<10	<10	<10	20	Flavivirus seropositive
7	04–1-06	80	<10	<10	20	<10	Zika virus seropositive
8	04–1-07	160	<10	<10	<10	<10	Zika virus seropositive
9	04–1-12	80	<10	<10	<10	<10	Zika virus seropositive
10	04–1-13	320	<10	<10	<10	<10	Zika virus seropositive
11	05–1-19	80	<10	<10	<10	<10	Zika virus seropositive
12	05–1-22	160	<10	<10	<10	<10	Zika virus seropositive
13	06–1-01	80	<10	20	<10	20	Zika virus seropositive
14	06–1-13	>320	<10	<10	10	<10	Zika virus seropositive
15	08–1-05	>320	<10	<10	<10	<10	Zika virus seropositive
16	09–1-22	>320	<10	10	20	<10	Zika virus seropositive
17	10–1-03	40	<10	<10	<10	<10	Zika virus seropositive
18	10–1-04	320	<10	<10	<10	<10	Zika virus seropositive
19	10–1-08	20	<10	<10	<10	<10	Zika virus seropositive
20	10–1-12	40	<10	<10	<10	<10	Zika virus seropositive
21	10–1-20	20	<10	<10	<10	<10	Zika virus seropositive
22	11–1-06	160	<10	<10	<10	<10	Zika virus seropositive
23	11–1-12	20	<10	<10	<10	<10	Zika virus seropositive
24	11–1-13	80	<10	<10	<10	<10	Zika virus seropositive
25	11–1-14	>320	<10	<10	<10	<10	Zika virus seropositive
26	11–1-16	40	<10	<10	<10	<10	Zika virus seropositive
27	11–1-22	20	20	10	80	<10	Flavivirus seropositive
28	12–1-01	20	<10	<10	<10	<10	Zika virus seropositive
29	12–1-19	20	<10	<10	<10	<10	Zika virus seropositive
30	12–1-21	40	<10	40	<10	<10	Flavivirus seropositive
31	13–1-01	40	20	<10	<10	<10	Flavivirus seropositive
32	13–1-10	160	<10	<10	<10	<10	Zika virus seropositive
33	13–1-14	80	<10	<10	<10	<10	Zika virus seropositive
34	13–1-22	160	<10	<10	<10	<10	Zika virus seropositive
35	14–1-12	160	<10	<10	<10	<10	Zika virus seropositive
36	16–1-12	160	<10	<10	<10	<10	Zika virus seropositive
37	16–1-21	80	<10	<10	<10	<10	Zika virus seropositive
38	18–1-02	80	<10	<10	<10	<10	Zika virus seropositive
39	18–1-06	160	<10	<10	<10	<10	Zika virus seropositive
40	19–1-01	160	<10	40	<10	<10	Zika virus seropositive
41	19–1-08	160	<10	<10	<10	<10	Zika virus seropositive
42	19–1-13	80	<10	<10	<10	<10	Zika virus seropositive
43	19–1-14	80	<10	<10	<10	<10	Zika virus seropositive

No.	Sample ID	PRNT$_{90}$ titer					Serostatus
		Zika virus	DENV-1	DENV-2	DENV-3	DENV-4	
44	19–1-15	80	<10	<10	40	<10	Flavivirus seropositive
45	19–1-16	160	<10	<10	<10	<10	Zika virus seropositive
46	20–1-02	160	<10	<10	40	<10	Zika virus seropositive
47	20–1-04	160	>320	>320	20	<10	Flavivirus seropositive
48	20–1-07	40	<10	<10	<10	<10	Zika virus seropositive
49	20–1-11	80	<10	<10	<10	<10	Zika virus seropositive
50	20–1-13	20	<10	<10	<10	<10	Zika virus seropositive
51	21–1-02	320	<10	<10	<10	<10	Zika virus seropositive
52	21–1-03	80	<10	<10	<10	<10	Zika virus seropositive
53	21–1-06	20	<10	<10	<10	<10	Zika virus seropositive
54	21–1-07	>320	<10	20	<10	<10	Zika virus seropositive
55	21–1-10	320	<10	<10	10	<10	Zika virus seropositive
56	22–1-04	160	<10	<10	<10	<10	Zika virus seropositive
57	23–1-04	80	<10	<10	<10	<10	Zika virus seropositive
58	23–1-14	20	<10	<10	<10	<10	Zika virus seropositive
59	23–1-15	160	<10	<10	<10	<10	Zika virus seropositive
60	23–1-20	>320	<10	<10	<10	<10	Zika virus seropositive
61	23–1-22	320	<10	<10	20	<10	Zika virus seropositive
62	24–1-02	40	<10	<10	<10	<10	Zika virus seropositive
63	24–1-16	40	<10	<10	<10	<10	Zika virus seropositive
64	24–1-21	80	<10	<10	<10	<10	Zika virus seropositive
65	25–1-06	80	80	<10	<10	<10	Flavivirus seropositive
66	26–1-03	320	<10	<10	<10	<10	Zika virus seropositive
67	26–1-14	20	40	<10	<10	<10	Flavivirus seropositive
68	26–1-22	20	<10	80	<10	<10	Flavivirus seropositive
69	28–1-03	80	<10	<10	<10	40	Flavivirus seropositive
70	30–1-01	320	<10	<10	<10	<10	Zika virus seropositive
71	30–1-12	320	<10	<10	<10	<10	Zika virus seropositive
72	30–1-22	160	<10	<10	<10	160	Flavivirus seropositive

*DENV, dengue virus; ID, identification; PRNT$_{90}$, plaque reduction neutralization test with neutralization defined as an ≥90% reduction in challenge virus PFUs.
